# Experts’ perspectives on the impact of visual impairment and comorbid mental disorders on functioning in essential life domains

**DOI:** 10.1186/s12888-024-05635-0

**Published:** 2024-03-18

**Authors:** Marjolein LA Onnink, Lisanne BA Teunissen, Peter FJ Verstraten, Ruth MA van Nispen, Hilde PA van der Aa

**Affiliations:** 1 Robert Coppes Foundation, Vught, The Netherlands; 2 Amsterdam UMC, Vrije Universiteit, Ophthalmology and The Amsterdam Public Health Research Institute, Amsterdam, The Netherlands

**Keywords:** Visual impairment, Autism spectrum disorder, Psychotic disorders, Obsessive-compulsive disorder, Antisocial personality disorder, Borderline personality disorder, Dependent personality disorder, Comorbidity, Functioning, Delphi

## Abstract

**Background:**

Visual impairment (VI) with comorbid mental disorders (MDs) are expected to have a major impact on people’s daily functioning, for which tailored support is needed. However, this has been barely investigated. Therefore, this study aimed to (1) determine the impact of VI and comorbid MDs on functioning in essential life domains, (2) gain insight into best-practices that are currently used to support this target group, and (3) determine strategies to optimize care in the future.

**Methods:**

A four-step qualitative Delphi method was used to obtain input from 31 Dutch professionals who work with this target group (84% female, mean age 46 years, on average 11 years of experience in working with the target group). The Self-Sufficiency Matrices were used to determine the impact on various aspects of daily living, for people with VI and (1) autism spectrum disorder, (2) psychotic disorders, (3) obsessive-compulsive disorder, (4) antisocial personality disorder, (5) borderline personality disorder, (6) dependent personality disorder.

**Results:**

Experts describe a frail and vulnerable population, in which the VI and MD often have a cumulative negative impact on people’s physical and mental health. People frequently experience anxiety, depression, fatigue and sleep disturbances. Also, many tend to neglect self-care and substance abuse is common. They often experience difficulty in trusting others while at the same time being dependent on them. Social interaction and relationships are complicated because of communication restrictions (e.g. no facial recognition) and social incompetence or withdrawal. Experts advise taking transdiagnostic factors into account, using evidence-based psychological treatment options based on an intermittent approach, and offering multidisciplinary care. They stress the importance of building trust, showing patience and empathy, stimulating empowerment, involving the informal network and building on positive experiences.

**Conclusion:**

VI and comorbid MD have a major impact on people’s daily functioning on a mental, physical, social and environmental level. This study provides insight into best-practices to support this target group. According to experts, more research is needed which could be aimed at investigating tailored diagnostic approaches and treatment options and include clients’ perspectives.

**Supplementary Information:**

The online version contains supplementary material available at 10.1186/s12888-024-05635-0.

## Background

Visual impairment (VI), according to the definitions of blindness and low vision proposed by the World Health Organization [1], substantially affects limitations in functioning and the ability to perform activities of daily living [2, 3]. People often experience reduced mobility [4] and difficulty with self-care [5]. Also, social network and support [6] and psychological well-being [7] are reduced, which seem to contribute to a spiral of increased frailty and vulnerability.

Mental disorders (MDs), e.g. mood disorders [7], developmental disorders [8], psychotic disorders [9, 10] and personality disorders [11] sometimes coexist in people with VI and may exacerbate perceived disability. Various directional hypotheses for the relationship between VI and MDs have been proposed. Studies indicate that VI significantly increases the risk of depressive and anxiety disorders [7], while other studies indicate that MDs can also increase people’s sensitivity to eye and vision problems [12, 13]. In people with autism spectrum disorder, a higher incidence of strabismus is found and several behavioural traits are common for both autistic and visually impaired populations [8]. In addition, schizophrenia can increase abnormal eye movements [14], reduced visual perception and processing disturbances [15], and borderline personality disorder has been associated with disturbances in visual perception [11]. Also, ageing may be a common underlying mechanism that causes both VI and MD in elderly patients, since the prevalence of VI significantly increases with age [16] and MDs are recognized as a major cause of disability and disease burden in older adults [17].

The combination of VI and MD (VIxMD) can have a cumulative impact on people’s daily functioning, as the VI can trigger problems due to the MD and vice versa [18]. For instance, people with borderline personality disorder often have unstable and intense interpersonal relationships, characterized by alternating between extremes of idealization and devaluation [19]. If these people also have VI, they may experience even more difficulties with maintaining relationships because of interpretation errors caused by missing nonverbal signals. Moreover, they may be dependent on others because of their VI while it is difficult for them to maintain a stable and healthy relationship with their loved ones or caregivers on whom they depend. Another example: people with autism spectrum disorder often have difficulties with estimating other people’s intentions, attributing feelings, thoughts, desires and intentions to others and themselves, and with explaining and predicting the behaviour of others [20–23]. People who also have VI may have additional difficulties with understanding other’s beliefs and interpreting other’s intentions because they cannot see and/or interpret social cues [18].

However, the impact of VIxMD on daily functioning seems to be barely investigated. Most studies focussing on the impact of VIxMD concern children and/or people with autism (Teunissen et al., unpublished results). For instance, Gense & Gense discuss career opportunities for children with VI and autism [24]. They report that people with this combination of problems have greater difficulties in terms of unemployment and underemployment than people with only VI or autism [24]. Additionally, some studies report on the impact of a psychotic disorder and VI on daily functioning. For instance, Moye (1998) suggests that VI contributes to misinterpretations of situations in daily live which are common in people with psychotic disorder [25].

Boessen et al. performed a descriptive study and scoping review to gain insight into the possible knowledge gaps and needs of professionals in order to optimize care for people with VIxMD [26]. It seemed that professionals were often not well equipped to work with the complex and diverse needs of this target group. For instance, most standard interventions for people with autism spectrum disorder consist of visual schedules, which are not accessible for people with VI [18]. Moreover, it was concluded that professionals working in different fields (e.g. low vision, mental health and elderly care) often lack specific knowledge about the impact of VIxMD on daily functioning [26].

Therefore, the aim of this study was to: (1) determine the impact of VIxMD on functioning in essential life domains based on the perspective of professionals, (2) gain insight into best-practices that are currently used to assist the target group and (3) determine strategies to optimize care for people with VIxMD in the future.

## Methods

### Design

A four-step qualitative Delphi method was used to obtain input from and establish consensus between Dutch professionals (‘experts’) between October 2019 and July 2020 [27]. In order to be able to involve as many experts as possible, their input was obtained via telephone and online questionnaires, reducing barriers in travel time and expenses. The study met the ethical standards of the Declaration of Helsinki (1964) and its later amendments, and the Medical Ethics Review Committee of Amsterdam University Medical Centres, location Vumc approved this study. All experts were informed and gave written consent to participate.

### Measurements

The Dutch version of the Self-Sufficiency Matrices (SSM) were used to determine the impact of VIxMD on various aspects of daily living. The SSM has been developed to map people’s functioning in 13 essential domains of life: (1) income, (2) work and education, (3) daytime activities, (4) household, (5) relations, (6) mental health, (7) psychical health, (8) addiction, (9) basic activities of daily life, (10) instrumental activities of daily live, 11) social network, 12) community involvement and 13) legal implications [28, 29]. The SSM shows acceptable psychometric features on reliability and validity [29]. The solid single factor structure and internal consistency of the scale were found to be excellent. In various studies, Cronbach’s alpha ranged between 0.85 and 0.89, indicating high internal consistency [29]. Moreover, the SSM has been shown to be highly consistent with the work process and culture of care systems for clients with multiple disorders in which an integrated, multidisciplinary approach is essential [28]. Many experts who participated in this study were already used to working with the SSM in their daily practice.

**Box 1:** Mental disorders that were the focus of study

Group	Mental disorder	Short definition
Developmental disorders	1) Autism spectrum disorder	Persons persistently experience challenges in social interaction, communication, and restricted/repetitive behaviours
Personality disorders	2) Antisocial personality disorder	Persons consistently show no regard for right and wrong and ignore the rights and feelings of others
	3) Borderline personality disorder	Persons show a long-term pattern of unstable relationships, strong emotional reactions and a distorted sense of self
	4) Dependent personality disorder	Persons show pervasive psychological dependence on others, characterized by fear and anxiety
Anxiety disorders	5) Obsessive-compulsive disorder	Persons experience uncontrollable, reoccurring thoughts (obsessions) and/or feel the need to repeatedly perform certain behaviours (compulsions)
Psychotic disorders	6) Schizophrenia	Abnormal thinking and perceptions, in which people experience delusions and hallucinations

### Procedure

Prior to the Delphi study, based on the input of experts working with clients with VIxMD (*n* = 4) and client representatives (*n* = 2), it was decided to focus on six MDs according to the DSM-V [19] that can be comorbid with VI, but did not or only marginally receive attention in previous studies (see Box 1). It was not feasible to target all MDs that coexist in people with VI. The experts and client representatives indicated that the impact of the combination of these specific MDs are high in people with VI and a lack of knowledge of these specific MDs was experienced.

To invite a large group of Dutch experts to provide their input for the Delphi study, key organizations and professionals, who work with this target group, were identified and contacted via personalized email invitations. They received an information letter, a consent form and an animated promotional brochure stating the importance of providing input on the impact of VIxMD on the life domains of the SSM. Also, they had the option to receive a digital voucher of €10.00 in appreciation for their time dedicated to this study. With the experts who provided written consent, an appointment for a telephone interview was scheduled.

The first step of the Delphi study consisted of a structured telephone interview to increase participants´ engagement, and to be able to fully explain the study procedure, answer questions and check inclusion criteria. Experts were asked to share basic background information (e.g. age, work experience and role). Based on their experience, they were asked to give their opinion on one or more MDs in people with VI. The interviews were recorded with permission of the participants. As preparation for the second step, professionals were asked to familiarize themselves with the first six domains of the SSM and think about the impact on daily functioning that clients may experience on these domains.

After four weeks, they were asked to fill out an online survey for which the online platform *Castor* was used (www.castoredc.com*).* In this survey, a short, recognizable, explanation of the first six domains of the SSM was provided and professionals were asked to provide their input on these domains. Open-ended questions were used to determine the impact of VIxMD on daily functioning and *do’s* and *don’ts* to use in practice were gathered. After each question, professionals were asked to explain their answers. They could also provide additional information if they wanted to. As preparation for the third step, the experts were asked to familiarize themselves with the last seven domains of the SSM and think about the impact on daily functioning that clients may experience on these domains.

After four weeks, they were asked again to fill out an online survey on these last seven domains and indicate per MD which domain they thought would have the most impact on daily functioning and explain why. Also, experts were asked to think about specific needs (i.e. knowledge, expertise, innovation) that they or other experts need to provide the optimal care tailored to the needs of the target population. After this step, the qualitative data were analysed in Atlas.Ti and a summary of the outcomes was written with general findings of the impact of VIxMD on daily functioning and specific findings for each MD individually.

In the final step, sixteen weeks later, the experts received this summary. In the final online survey, they were asked what they missed or had not recognized in this summary. And which three domains on the impact on daily functioning per MD they thought were most relevant.

### Participants

Experts from three low vision service organizations in the Netherlands (i.e. the Robert Coppes Foundation (RCF), Royal Dutch Visio and Bartiméus) were invited to participate in this study via e-mail and internal websites of their organizations. Experts from other Dutch healthcare organization (i.e. within elderly care and mental health care) who work with people with VIxMD were also invited to participate via e-mail and the online platform *LinkedIn*. Email addresses were obtained via the vast network of the authors. Also, experts were recruited via the online platform www.psyvisnet.nl. This is an online community of practice, which has been developed by and for professionals who work with people with VIxMD to share best practices, knowledge and expertise and learn from each other.

Experts were included if they met the following criteria: (1) being a health care professional (i.e. psychiatrist, psychologist, behavioural scientist, social worker or group counsellor), (2) treating or counselling people over the age of 18, with VI and one or more of the previously mentioned MDs: antisocial personality disorder, borderline personality disorder, dependent personality disorder, autism spectrum disorder, obsessive-compulsive disorder and psychotic disorders, and (3) working with this target group for a minimum of one and a half year.

In total, 31 experts participated in the study. Most of them were counsellors (35%) and behavioural scientists or psychologists (35%, see Table 1). On average they were 46 years old, 84% were female and they had 11 years of experience in working with the target group. Most of them gave their professional opinion on the impact of VI in combination with autism spectrum disorder (74%), followed by borderline personality disorder (65%), dependent personality disorder (45%), psychotic disorders (45%), antisocial disorder (19%), and obsessive compulsive disorder (13%).

## Results

### General findings

Several themes concerning daily functioning were similar across VIxMD subgroups. These general themes could be categorized by: (1) quality of life (psychological, physical, social and environmental) and (2) quality of care related topics. Supplementary Tables S1 and S2 provide an overview of the general findings on the impact of visual impairment (VI) and mental disorders (MD) on quality of life and quality of care, respectively. Next, the general findings concerning quality of life and quality of care will be presented by theme.

### Quality of life: psychological

According to the experts, people with VIxMD in general seem to frequently experience anxiety, depression and loneliness and they often have low self-esteem. Moreover, some of them experience little pleasure in life and are often disappointed in others and themselves. In addition, they indicate that some people seem to have great difficulty adapting to or accepting their problems; they tend to deny their hardship and experience great difficulty in asking for help (from professionals or others). Many people lack the proper coping skills and/or cognitive abilities to deal with this. An expert wrote:

Acceptance is difficult when people lack self-understanding and taking initiatives that lead to a positive life-attitude.

Some people even experience a reduced sense of time due to their disabilities, i.e. their perception of the duration of the indefinite and unfolding of events seems to be disturbed. As for learning abilities, VI already requires (complex) knowledge for clients in order to function on a basic level in many areas. An additional MD may negatively affect this learning ability.

**Table 1:** Participant characteristics (*n* = 31)

Participant characteristic		N (%)	Mean (SD)	Median [Range]
Age			46 (12)	46 [25–62]
Female gender		26 (84%)		
Years of experience in working with the target group			11 (8)	10 [1.5–30]
Role*	(Group) Counsellor	11 (35%)		
	Behavioural scientist/Psychologist	11 (35%)		
	Social worker	6 (19%)		
	Scientist	1 (3%)		
	Psychiatrist	1 (3%)		
	Unspecified**	8 (26%)		
Gave professional opinion on*	Autism Spectrum Disorder	23 (74%)		
	Borderline personality disorder	20 (65%)		
	Dependent personality disorder	14 (45%)		
	Psychotic disorders	14 (45%)		
	Antisocial disorder	6 (19%)		
	Obsessive compulsive disorder	4 (13%)		

*Multiple answers possible

**Some experts selected ‘unspecified’ when they did not recognize their profession in one of the specified roles

Experts indicate that more research is needed on loneliness, self-image, self-confidence and sense of mastery in this population based on clients’ perspectives.

### Quality of life: physical

In general, healthy living can be very difficult for people with VIxMD. It is difficult for them to recognize physical problems, due to which neglect of self-care may occur. Self-medication seems to occur regularly, just as excessive drinking and drug abuse. In addition, living with VIxMD requires a lot of energy (sometimes aggravated by the use of medication) leading to severe fatigue and many seem to experience sleep disturbances.

### Quality of life: social

Due to their VI, people are dependent on others for many activities in daily life. However, their MD may complicate this dependency. Many clients have difficulty trusting others and estimating who can be trusted. An expert explained:

Trust in other people is often already seriously damaged, often there is a large deal of distrust, therefore, building a relationship takes a lot of time.

In addition, many people have difficulties with day-time activities and community participation because there are less opportunities. Many people have no or only a small network. People can have more difficulty building and maintaining social relationships. An expert stated:

A lack of good social contacts has a large impact on quality of life. I am often shocked by the few social contacts that our clients usually have. Also, they often do not have a partner.

Due to a high risk of misinterpretation and miscommunication as a result of the VIxMD, conflicts and/or relational problems will arise more quickly. Some people have limited social skills because they cannot see role model behaviour from others. With regard to stigmatization, it seems that resilience is warranted. An expert noted:

Due to stigmatization, people need to be extra resilient in order to feel social trust in case of a desire to have children for instance.

Experts indicate that more research is needed on relationships and intimacy in this population based on individual client perspectives.

### Quality of life: environment

A safe environment, tailored to the VI, seems to be crucial for people with VIxMD. Assisted living, e.g. in an institution, can be supportive because problems can be detected more quickly. However, conflicts between roommates may also arise frequently. Paid employment is often not possible. An expert explained:

It is very difficult for them to get work or to function within a work setting together with colleagues and be in contact with others. […] It is often problematic because of incomprehension and limited people skills.

Employers often underestimate the impact of VIxMD and the workload is often too high. Some clients also seem to have trouble working together with others. An appropriate work environment is essential, in which a workplace ‘buddy’, who supports the client with his/her work, can be very beneficial for all involved. In addition, financial problems seem to occur regularly. Many have relatively little income because they often depend on allowances or benefits. Moreover, they also often experience difficulties maintaining an overview of their finances and take impulsive decisions more quickly. There is a risk that the environment will exploit this vulnerability. Concerning instrumental activities of daily living, people can experience large difficulties running a household independently. An expert noted:

Sometimes people have excessive amount of stuff and they can have difficulty interpreting information (e.g. letters from agencies).

A final topic concerning quality of life (environment) was criminal offenses. People are less likely to get involved in situations where criminal offenses are committed; possibly because the protective influence of assisted living. When one does come into contact with the law, it is often because of externalizing behaviour.

### Quality of care

Due to the multi-morbidity, it is often difficult to properly diagnose the MD that people are suffering from. Therefore, it can be important to use psychiatric observations in order to identify problems at an early stage. An expert stated:

The impact of not being able to properly identify problems and where it comes from has great consequences. Clients feel misunderstood, experience failure and disappointments.

Experts indicate that more research is needed on transdiagnostic factors and tailored diagnostic approaches to optimize care for people with VIxMD. They suggest also looking at resilience and success factors. After diagnosis, timely use of treatment and a stable treatment team are recommended. Also, multidisciplinary cooperation (i.e. between low vision services, general practitioners, addiction care, mental healthcare, mentors etc.) is recommended to offer tailored support with a proper focus on both the VI and the MD. Unfortunately, many experts experience a lack of such collaborations. In addition, an intermittent approach (i.e. offering interventions at times that a clients is more stable) is desirable, in which it is recommended to make use of the few evidence-based psychological treatment options that are available tailored to the needs of people with VI. Attention should be paid to both the VI and the MD, and both to the individual client and the group dynamics (in case a client is living in a residential setting). During treatment it is considered important that people experience a sense of mastery and that professionals leave the responsibility of progress with the client. Because clients are vulnerable, a good balance needs to be sought between protecting/advising and letting clients make their own decisions. It is recommended to involve both the client as well as the informal network (i.e. family and friends), roommates and third parties (e.g. work) when providing psychoeducation. Moreover, experts advised to support clients in expanding and maintaining their informal network. Also, it is advised to look for the best possible way of communication. For instance, (technological) tools can provide adequate support in some cases, in addition to regular counselling moments. Still, treatment adherence is a concern. A relationship of trust and an empathetic, open attitude seem to be crucial in order to provide adequate support. Clarity, setting boundaries and realistic expectations are important. Offering opportunities, finding positive influences and focussing on success experiences can contribute positively. In this context, the ‘presence-approach’ is often used, i.e. taking time to get to know clients and their environment deeply and strive to affirm the fundamental dignity of clients. While this approach is not directly focusing on problem-solving, it may lead to problem-solving over time. Still, experts indicate that much more research is needed on tailored support and treatment options for people with VIxMD.

### Findings per VIxMD, ordered by DSM-V [19]

Several themes were found for specific VIxMD subgroups which could also be categorized by these two topics (quality of life and quality of care), and included best practices and strategies to optimize care. Supplementary Tables S3 and S4 provide an overview of findings per mental disorder (MD) in combination with visual impairment (VI) on the impact on quality of life and quality of care, respectively.

### Autism spectrum disorder

#### Quality of life

Specifically for autism spectrum disorder in combination with VI, experts indicate difficulty in social relationships. An expert explained that many people with congenital blindness exhibit behaviours that correspond to autism because they cannot observe social contexts. Moreover:

Both disorders are disadvantageous to maintain social contacts: due to less developed social skills and the fact that communication can be unclear.

Also, people can get into unexpected situations because of their VI which is extremely difficult due to their autism spectrum disorder. An expert explained:

Other people often want to help people who are blind but do not verbalise or communicate what they are doing, leaving the blind person unaware of what is happening. This is a huge additional obstacle for people with autism.

Developing skills and competences (e.g. concerning household, finances and personal care) are crucial in order to live as independently as possible. Keeping sight of the big picture is very important for people with autism in developing such skills, however, due to their VI this is complex because they cannot learn from the context and visual information.

### Quality of care

Professionals and significant others tend to overestimate the capabilities of people with VI and autism spectrum disorder. An expert explained:

Many people think that the client can apply an advice to another situation, but they can’t.

Professionals should be aware that asking for help can also be very difficult for these clients. Psychoeducation (i.e., discussing symptoms of MD, possible causes, treatment options, prevention and coping strategies) seems to be very important. Moreover, learning (social) skills seems to be crucial in treatment, for which it is recommended to let people build on positive experiences.

### Psychotic disorders

#### Quality of life

People with a psychotic disorder and VI, often seem to experience anxiety and depression. They often cannot take comfort in familiar things or environments that can put thoughts into perspective. An expert explained:

Not being able to see what is happening around you makes it difficult to participate in the world around you. You just have to trust that it is okay. This is extremely difficult for people who experience psychoses.

People often end up in a situation of neglect and often experience a deregulation of their circadian rhythm, i.e. the internal process that regulates the sleep–wake cycle. Increased anxiousness and distrust may lead to loneliness and social isolation. An expert stated:

If distrust is part of your thinking, it will often lead to uncomfortable communication, perhaps even avoidance or exclusion.

Living in a residential setting and even the VI itself can be a protective factor for these clients in limiting their exposure to (physical and mental) injury. An expert explained:

Sometimes it can be sort of an advantage that clients are less mobile due to their visual impairment. A client of mine caused damage to his apartment during a psychosis, but couldn’t go outside to get money. As a result, the financial damage was not that high.

Another expert advised:

Don’t respond too much to the behaviour that thoughts and sounds evoke, but put it more into perspective and provide distractions.

### Quality of care

Especially for these clients, a good balance needs to be sought between the professional roles as protector or advisor (especially when clients experience psychotic symptoms) and, if possible, allowing them make their own decisions. It is very important for these clients to feel a sense of mastery or control over their lives. In addition, building a trustful relationship is very important. An expert advised:

A stable team of professionals works best. If you see a client a lot and have known him for years, small changes may already be noticeable.

Psychotic symptoms should be identified at an early stage and it is important to anticipate on possible problems in the future. Experts explained:

The impact of crisis care can be enormous and in itself can cause trauma due to the elevated feelings of insecurity and Make agreements with the client for relapses.

### Obsessive-compulsive disorder

#### Quality of life

An obsessive-compulsive disorder in people with VI often has a large impact and may lead to excessive anxiety levels. An expert explained:

I think the visual impairment reinforces the fear because people can’t see what is happening when something goes wrong in their minds.

As a consequence, the private living space seems to be the only safe place for them. Moreover, the anxiety costs a lot of energy. An expert wrote:

The impact is big, this combination of problems costs a lot of time and energy, the level of suffering is often high.

Sometimes daily tasks require so much energy that there is hardly any time left for social contacts. The (limited) social network that is available often has to adapt completely to the client’s needs. As for physical health, there seem to be extremes. Some clients are extremely concerned with their health. An expert stated:

Usually there is enough (or excessive) attention for GP visits, food and drinks, medication etc.

On the other hand, some people have little time and space to take care of themselves. Work can be problematic and cause imbalance, because they do not receive the same level of support at work as they do at home, adapted to their MD and VI.

#### Quality of care

In many cases it is advised to refer these clients to an experienced mental healthcare specialist. Sometimes medication can be necessary. Additionally, having a lot of patience and empathy seem to be important, and using a positive approach. An expert added:

If there are agreements on certain compulsions between the client and others [professionals], try to comply with them [as professional]”. Lastly, it is advised to pay attention to managing clients’ expectations of others in psychoeducation.

### Antisocial personality disorder

#### Quality of life

As a result of the antisocial personality disorder, clients often have a small or unreliable network, on whom they are dependent because of their VI. Clients sometimes misuse others for their own interest. An expert stated:

Clients build relationships mainly based on their own interest, often there is little real reciprocity and they abuse the relationship, which ultimately ends the relationship. Because of the visual impairment, it is more difficult to estimate what the consequences of their actions are.

In addition, the combination of VI and antisocial personality disorder can make it more difficult to find appropriate day-time activities. Also, financial problems seem to occur more often.

#### Quality of care

Especially with these clients, it seems important that professionals ensure their own safety. An expert explained:

Try to create a safe environment for professionals, consider visiting a client with a colleague if necessary.

After a while it can be more difficult for a professional to empathise with the client due to his/her behaviour. It is important to keep in mind that these clients are still very vulnerable and need help. An expert noted:

The visual impairment may evoke pity, which may lead to a more indulgent approach.

Another expert advised to arrange supervised administration, possibly as a condition in order to get into assisted living. This may enable professionals to maintain a neutral position concerning finances. Clients are often not open to feedback. It is advised to leave the client fully responsible for his/her own actions and deeds.

### Borderline personality disorder

#### Quality of life

People with borderline personality disorder often engage in idealization and devaluation of others, alternating between high positive regard for people and great disappointment in others. The VI makes them dependent on others, which in some cases reinforces their admiration for others as well as their dislike after a disappointment. This may lead to strong emotional reactions, which cost a high amount of energy. Moreover, interpretation errors occur even more often due to both the borderline personality disorder and the VI. Also, the manipulative behaviour that people show can be complicated because people with VI cannot properly assess what their environment perceives about their behaviour. An expert explained:

Both disorders influence each other. People with borderline personality disorder often try to manipulate their environment. However, I notice that clients with visual impairment regularly fail to do so because they are found out. Simple example: someone says he lately took out the garbage, but one can see the garbage bags are still in the garden.

Often people have no or only a small network. An expert wrote:

Sometimes the network of these people consists only of professionals.

Living in a large, anonymous and/or relatively unsafe residential setting seems to increase the feeling of insecurity among these clients. There seems to be an increased risk of dissatisfaction with the housing situation (and possibly discontinuity thereof). An expert advised:

If someone has a place to live, try to hold on to it. Try to improve the conditions over there, because moving causes a lot of disturbance and probably the same problems.

Concerning finances, distrust can be a complicating factor in accepting help. In addition, it seems that people make impulsive decisions, sometimes to favour other people, without an adequate assessment whether the financial resources are sufficient.

### Quality of care

Gaining trust and keeping it, are exceptionally difficult in supporting these clients, because of their emotionally unstable personality and their VI (non-verbal signals are not picked up). Slowly building a relationship of trust and having a neutral attitude seem crucial in order to be able to do so. An expert explained:

Take a lot of time to build trust, try to not judge clients’ behaviour. If you go too fast, the client will drop out and experience you as a meddler.

It is advised to explicitly make clients responsible for their own choices and behaviour, since they are inclined to hold others responsible.

### Dependent personality disorder

#### Quality of life

Due to their VI, people are dependent on others for many things in daily life. This may reinforce the difficulty that people with a dependent personality disorder have in becoming emotionally overdependent on others. An expert explained:

People with a dependent personality disorder have difficulty with autonomy. The VI reinforces this: sometimes they have to depend others.

Another expert added:

Due to progression in visual problems over time, their dependency is also increasing and may sometimes be used as an excuse.

In addition, because of their VI it can be more difficult for clients to gain and maintain social contacts, although they experience a great desire for it. There is a chance that the network will only consist of professionals. Many of these clients live in residential settings. An expert explained:

This [living in residential settings] facilitates their lives considerably, but their dependency on others is automatically greater. This sometimes reinforces the problem and strengthens their belief that they are not able to do it on their own.

### Quality of care

It is recommended to stimulate clients’ sense of mastery. A positive approach in which people are able to have successful experiences seems to be important. An expert suggested:

Connect to someone’s capabilities and what he/she can handle. With small steps let someone achieve successes… and look back on these successes together.

Lastly, Table 2 provides an overview of the psychological, physical, social and environmental client characteristics that influence the impact of VIxMD on daily functioning, according to experts.

**Table 2:** Client characteristics that influence the impact of VIxMD on daily functioning

Psychological	Physical	Social	Environmental
• Degree of experienced negative emotions (i.e. depression, anxiety) • Understanding of the disease and oneself • Experiencing a sense of mastery or control • Coping skills • Personality • Acceptance of the VI and MD	• Severity and time of onset (congenital or acquired) of the VI • Severity of the MD and possible comorbidities • Cognitive ability • Overall health status	• Social skills • Social network • Social support	• Day time activities and daily structure • Attitude of third parties, such as employers • Availability of professional support, trust in them

## Discussion

In this study we investigated the impact of VIxMD on daily functioning. We asked experts about the impact of visual impairment in combination with mental disorders on people’s daily functioning, with an equal focus on both. We focussed on autism spectrum disorder, antisocial personality disorder, borderline personality disorder, dependent personality disorder, obsessive-compulsive disorder and psychotic disorders. Experts who work with this severely physically and mentally disabled target group, describe a frail and vulnerable population that needs tailored support from experienced professionals.

People with VI frequently experience anxiety, depression [7] and fatigue [30], especially those who have difficulty adapting to their VI [31, 32]. If these people also have a comorbid MD, they are even more likely to be suffering from these psychopathological symptoms, which are inherent to their MD diagnosis as well [19]. Experts especially associate borderline personality disorder, obsessive compulsive disorder and psychotic disorders with anxiety and depression in this population, which is in line with current literature [33–35]. Moreover, experts indicate that for clients with obsessive compulsive disorder and VI, daily tasks and coherent anxiety may often lead to extreme (mental) fatigue, which is supported by the literature [30, 36].

Healthy living can be very difficult for people with VIxMD. Experts indicate that the physical health of clients is often low due to neglect of self-care, self-medication, alcohol and drug abuse, which is confirmed by a study by Najt et al., 2011 [37]. Moreover, sleep disturbances and deregulation of a person’s circadian rhythm are prevalent in people with VI, especially in those with a loss of light perception [38]. Comorbid MDs (especially psychotic disorders according to experts) can seriously aggravate the sleep–wake cycle with a major impact on people’s physical and mental health [39].

Socially, people with VIxMD often have difficulty trusting others while they are dependent on them for many activities in daily life. In addition, the VI affects communication that is critical for social interaction, such as face recognition and the ability to distinguish facial expressions [40]. Communication challenges and other aspects of life with VI can threaten social participation, thereby possibly isolating the person, negatively affecting their health and reducing their degree of engagement with society [6]. Comorbid MDs may have a cumulative impact on this social withdrawal. Especially people with visual impairment and autism spectrum disorder, antisocial personality disorder and dependent personality disorder experience extreme difficulty in social interactions and relationships [19]. Moreover, people need to be extra resilient with regard to social stigmatization (i.e. prejudice and discrimination) concerning their VI and MD, which may cause feelings of shame, hopelessness and isolation [41].

Participation in (voluntary) work and learning activities are extremely challenging for people with VIxMD, due to the tremendous obstacles they face. Of the working-age population with VI, only one in three persons has a paid job, and they often experience high levels of stress, fatigue and dissatisfaction [42]. The VI requires (complex) knowledge, aided by assistive technology, in order to function on a basic level. Additional MDs complicate this due to reduced social skills and increased risks of mental and cognitive impairment [43]. An appropriate workplace environment seems essential, in which clients receive similar levels of support as they do at home.

According to experts, the severity of the visual impairment and the time of onset (congenital or acquired) are important characteristics that may influence the impact of VIxMD on daily functioning. Because of the comorbidity of physical and mental disabilities, experts indicate that it is often difficult to properly diagnose the MD that people are suffering from. Moreover, people who have one MD are likely to meet criteria for additional MDs at rates far exceeding what would be expected based on prevalence estimates [44]. Therefore, experts suggest making use of observational diagnostics by also taking transdiagnostic factors into account. Examples of such transdiagnostic factors in this population are anxiety, depression, mental fatigue and sleep disturbances, which can be triggered by the VI but may also indicate an underlying MD. More research is needed that build on these suggestions to be able to offer tailored diagnostic approaches. Also, it would be desirable to focus on individual VIxMDs so more in-depth knowledge can be gained.

Experts recommend using evidence-based psychological treatment options tailored to people with VI to reduce anxiety, depression and fatigue in this population, such as self-management programmes, behavioural activation or stepped-care [45, 46]. However, according to the experts this should be offered based on an intermittent (or discontinuous) approach, during which the active interventions are only offered at times that a client is stable and able to follow the intervention. Also, the cognitive abilities of clients to follow these interventions should be taken into account. Other options that may be more suited to some clients’ needs and abilities are mentalization based therapy [47] or mindfulness-based stress reduction [48]. Still, experts indicate that much more research is needed on tailored support and treatment options for people with VIxMD.

In addition, experts recommend multidisciplinary care in which e.g. low vision service providers, general practitioners, addiction care workers and mental healthcare specialists work together to offer tailored support with a focus on both the VI and MD. Currently, experts experience a lack of such collaborations while individual professionals lack specific knowledge on either the VI or MD [26]. A stable treatment team should be appointed offering comprehensive multidisciplinary care that addresses the client’s health and other needs. Such collaborations have proven to be very effective in clients with chronic diseases and/or complex care needs [49]. During treatment, experts stress the importance of building trust, showing patience and empathy, increasing the clients’ sense of mastery or control over their lives by stimulating empowerment, involving the informal network (e.g. family, friends, roommates, colleagues) and building on positive experiences.

## Strengths and limitations

Several experts with many years of experience in working with the target population gave their professional opinion on the impact of VIxMD on daily functioning, best-practice methods and strategies to optimize care. The explorative qualitative Delphi design allowed for in-depth information on this fragile population and motivates follow-up research. However, we did not incorporate the perspective of clients themselves and included only six mental health conditions based on the knowledge gap experienced by experts and client representatives concerning these specific MDs. Moreover, most experts worked in one of the three (collaborating) organizations for people with VI, so there was no control group of experts who use different approaches. Future studies may focus on examining clients’ perspectives and incorporating additional MDs that can be comorbid with VI, such as major depressive disorder, anxiety disorders [7] and post-traumatic stress disorder [50]. Also, the impact of the severity and onset (congenital or acquired) of VI and a specific focus on individual MDs may be explored.

## Conclusions

The combination of VI and MD has a major impact on people’s daily functioning on a mental, physical, social and environmental level. Several important transdiagnostic domains on daily functioning were found, while also specific domains per VIxMD were found. Experts describe a severely physically and mentally disabled target group that needs tailored support from experienced professionals. On a transdiagnostic level, they advise offering multidisciplinary care, taking transdiagnostic factors into account, and using evidence-based psychological treatment options based on an intermittent approach. They stress the importance of building trust, showing patience and empathy, involving the informal network, stimulating empowerment, and building on positive experiences during treatment. More research on this complex combination of problems is necessary, which could be aimed at investigating tailored diagnostic approaches and treatment options and including the client’s perspective on e.g. loneliness, relationships and intimacy, self-image, self-confidence and sense of mastery and control.

### Electronic supplementary material

Below is the link to the electronic supplementary material.

**Supplementary Material 1: Supplementary tables.** General findings and specific findings per mental disorder

## Data Availability

The datasets are available from the corresponding author on reasonable request.

## Abbreviations

VI: Visual Impairment
MD: Mental Disorder
VIxMD: Visual Impairment and Mental Disorder
