# Supplementary material for: Experts’ perspectives on the impact of visual impairment and comorbid mental disorders on functioning in essential life domains
Source: BMC Psychiatry. 2024 Mar 18;24:209. doi: 10.1186/s12888-024-05635-0 (PMC10946163; doi:10.1186/s12888-024-05635-0)
Supplement: Supplementary file 1 — Supplementary Material 1: Supplementary tables. General findings and specific findings per mental disorder [file 12888_2024_5635_MOESM1_ESM.pdf]

Supplementary Table S1 Overview of the general findings on the impact of visual impairment (VI) and mental disorders (MD) on quality of life

**Psychological**	Mental health	Susceptible to feelings of anxiety, depression, loneliness, low self-esteem, insecurity, little pleasure, disappointed in self/others, motivational problems
	Acceptance	Difficulty adapting to or accepting problems, deny hardship
	Asking for help	Difficulty asking for help from professionals or others
	Developing skills	Negatively affected learning abilities, lack of proper coping skills and/or cognitive abilities to deal with problems
	Time management	Reduced sense of time
**Physical**	Healthy living	Challenges in maintaining a healthy lifestyle and self-medication, susceptible to excessive drinking and drug abuse
	Recognizing	Difficulty in recognizing physical problems, neglect of self-care
	Fatigue	Susceptible to experience fatigue in daily life, sensory overload, exceeding one's personal capabilities, difficulties concerning the circadian rhythm, sleep disturbances
**Social**	Day-time activities	Challenges with day-time activities and community participation, reduced areas of interest
	Dependency	Complexity in dependency on others
	Trusting others	Difficulty trusting others and estimating who can be trusted
	Social netwerk	No or only a small network, high burden on the small social network
	Social interactions	Difficulty building and maintaining social relationships, risk of misinterpretation and miscommunication, conflicts and/or relational problems, limited social skills, sub-assertiveness, stigmatization
**Environmental**	Living environment	Limited adequate housing options, assisted living can be supportive but carries the risk of conflicts and burdening roommates
	Work environment	Paid employment often not possible, overestimation by employer, high workload, uncertainties about others' expectations, trouble working together with others
	Finances	Financial difficulties, low income, difficulty maintaining an overview, impulsive decisions, vulnerability to exploitation
	Instrumental ADL	Difficulties running a household independently, an excess of possessions, difficulty interpreting (formal) information
	Criminal offenses	Less likely to engage in criminal offenses

Supplementary Table S2 Overview of general findings on the impact of visual impairment and mental disorders on quality of care

Findings	Advice
- Difficult to properly diagnose MD's<br>- Lack of multidisciplinary cooperation<br>- Treatment adherence is a concern	**Professional attitude**<br>- Presence approach: take time to get to know clients and their environment and strive to affirm the fundamental dignity of clients<br>- Empathetic, open attitude to create relationship of trust<br>- Provide clarity, set boundaries, create realistic expectations<br>- Offer opportunities, find positive influences, focus on success experiences<br>- Find a balance between protecting/advising and letting clients make their own decisions<br>- Leave the responsibility of progress with the client<br><br>**Practical considerations**<br>- Pay attention to both the VI and the MD<br>- Pay attention to the individual client and possible group dynamics<br>- Aim for multidisciplinary collaboration<br>- Use an intermittent approach (offering interventions at times that a clients is more stable)<br>- Use psychiatric observations in order to identify problems at an early stage<br>- Aim for timely use of treatment and a stable treatment team<br>- Use tailored evidence-based psychological treatment options, if available<br>- Help clients develop a sense of mastery<br>- Support clients in expanding and maintaining their informal network<br>- Involve clients and informal network in psychoeducation<br>- Look for the best possible way of communication and support (including technical tools)

Supplementary Table S3 Overview of findings per mental disorder (MD) in combination with visual impairment (VI) on the impact on quality of life

**VI & Autism spectrum disorder**	Developing skills	Difficulty in developing general skills and competences
	Recognizing	Difficulty in recognizing physical problems in self
	Social interactions	Difficulty in initiating and maintaining social relationships and in unexpected situations
**VI & Psychotic disorder**	Mental health	Susceptible to distrust, anxiety, depression and difficulty finding comfort in familiar things that can put thoughts into perspective
	Fatigue	Susceptible to experience fatigue in daily-life, deregulation of circadian rhythm
	Day-time activities	Susceptible to anxiety about being outside of one's own residence
	Social network	Susceptible to loneliness, social isolation
**VI & Obsessive-compulsive disorder**	Mental health	Susceptible to excessive anxiety levels, high distress
	Healthy living	Extremely concerned with health or little time and space to take care of health
	Fatigue	Anxiety costs a lot of energy, little time for social contact or day-time activities outside daily life
	Social network	Network often adapted completely to client's needs
	Work environment	Disquiet caused by work
	Living environment	Private living space the only safe place
**VI & Antisocial personality disorder**	Social network	Small or unreliable network
	Social interaction	Misuse others for own interest, network may tolerate more antisocial behavior due to someone's VI
**VI & Borderline personality disorder**	Social network	No or only a small network
	Social interaction	Susceptible to interpretation errors in social communication, strong emotional reactions to people on whom they are dependent, manipulative behaviour can be complicated
	Living environment	Living settings can increase the feeling of insecurity, risk of dissatisfaction with/discontinuity of housing situation
**VI & Dependent personality disorder**	Social network	Susceptible to becoming emotionally overdependent on others, network may consist only of professionals
	Living environment	More often living in a residential environment, increased potential for hospitalization

Supplementary Table S4 Overview of findings per mental disorder (MD) in combination with visual impairment (VI) on the impact on quality of care

	**Advice**
**VI & Autism spectrum disorder**	- Be aware of risk of overestimating capabilities of clients by professionals and significant others<br>- Psychoeducation can be very important<br>- Pay attention to clarity, structure and learning (social) skills<br>- Let clients build on positive experiences<br>- Be aware that asking for help can be difficult<br>- Organize a buddy system in the working environment
**VI & Psychotic disorder**	- Identify psychotic symptoms at an early stage<br>- Anticipate on possible problems in the future, make agreements for relapses<br>- A good balance needs to be sought between the professional roles as protector or advisor (especially when clients experience psychotic symptoms)<br>- Help clients develop a sense of mastery<br>- Build a trustful relationship
**VI & Obsessive-compulsive disorder**	- Have a lot of patience and empathy, use a positive approach<br>- Adhere to agreements with the client<br>- Pay attention to managing clients' expectations of others in psychoeducation<br>- Referring to an experienced mental healthcare specialist may be the optimal choice<br>- Medication can be necessary
**VI & Antisocial personality disorder**	- Ensure professionals' own safety<br>- Leave clients fully responsible for their own actions and deeds<br>- Keep in mind clients are still very vulnerable and need help<br>- Arrange supervised administration, potentially as a requirement for assisted living
**VI & Borderline personality disorder**	- Use presence approach<br>- Slowly build a relationship of trust<br>- Maintain a neutral attitude and strive to avoid judging the client's behaviour<br>- Make clients responsible for their own choices and behaviour
**VI & Dependent personality disorder**	- Stimulate clients’ senses of mastery<br>- Use a positive approach<br>- Let clients have successful experiences and look back on these successes<br>- Connect to clients' capabilities and assess what the client can handle
